# Cross-Sectional Survey among General Population Regarding Knowledge and Attitude toward Antibiotic Usage in Western Saudi Arabia

**DOI:** 10.3390/pharmacy9020098

**Published:** 2021-05-01

**Authors:** Syed Faisal Zaidi, Muhannad Wael Baroom, Adil Ibrahim Hanbashi, Abdulrahman Abdulaziz Alkhaibari, Ahmed Omar Yahya, Muath Alsalmi, Rakan Alotaibi, Abdulaziz Nagro, Muhammad Anwar Khan, Asim Muhammed Alshanberi

**Affiliations:** 1 College of Medicine, King Saud bin Abdulaziz University for Health Sciences, Jeddah 21423, Saudi Arabia; mwbaroom@hotmail.com (M.W.B.); adil99-9@hotmail.com (A.I.H.); abduh-220@windowslive.com (A.A.A.); ahmed.rs40@gmail.com (A.O.Y.); alsalmi009@ksau-hs.edu.sa (M.A.); alotaibim007@ksau-hs.edu.sa (R.A.); nagro024@ksau-hs.edu.sa (A.N.); khana@ksau-hs.edu.sa (M.A.K.); 2 Department of Pharmacology, School of Medicine, Batterjee Medical College for Sciences and Technology, Jeddah 21442, Saudi Arabia; amshanberi@uqu.edu.sa; 3 Department of Community Medicine and Pilgrims Health Care, Umm Alqura University, Makkah 24382, Saudi Arabia

**Keywords:** antibiotics, knowledge, attitudes, usage, resistance, public’s awareness, Jeddah, Saudi Arabia

## Abstract

Background: Antibiotic resistance is a threatening issue to human wellbeing and an obstacle in the treatment process of many life-threating illnesses. This study aims to assess the knowledge and attitudes toward antibiotic usage among the general population in Jeddah, Saudi Arabia. Methods: A self-administered cross-sectional survey of 460 participants was distributed among the general population in Jeddah in the form of a validated questionnaire. Sample size was calculated to be 460 adults of either gender. Descriptive and inferential statistical analyses were performed using the Statistical Package for Social Sciences. Results: the age of more than half of participants (55.6%) was 18–30 years old, followed by the age group 31–40 years old (26.6%), with the smallest age group >60 years old (1.9%). More than two thirds of participants were male (69.5%), while 131 were female, accounting for 30.5%. Almost one third of participants had poor knowledge about antibiotics (30.5%), 51.0% had used antibiotics without any prescriptions, 54.6% of participants thought antibiotics could be used to treat viral infections, and 55.1% thought it was acceptable to stop taking antibiotics if symptoms start to improve. In addition, 49% believed that taking antibiotics would help them get better more rapidly when suffering from the common cold. Some personal characteristics were significantly associated with the public’s knowledge (e.g., age, education, and monthly income) and their attitudes (e.g., monthly income). Conclusion: Findings revealed a low level of knowledge on the use of antibiotics among the general public in Jeddah. This study signifies the need for improvement in the public’s knowledge and enhancement of their attitudes toward proper utilization of antibiotics.

## 1. Introduction

In the 21st century, one of the issues that threatens human wellbeing is the antibiotic resistant phenomenon [1]. The pharmaceutical industry is working hard to cope with the challenge of developing new antibiotics with more availability, broader spectrums, and better effectiveness against resistant bacteria, however, the emergence of superbugs is still a humongous challenge the world over. [2]. In addition, 47% of all antibiotics that are used belong to the penicillin group [3]. It has been found that in primary care clinics more than two thirds of upper respiratory tract infections (URTIs) are treated with antibiotics [4]. The vast majority of URTIs are caused by a viral infection, for which the use of antibiotics is inappropriate and has no clinical benefit [4,5,6].

Inappropriate behavior and consumption of antibiotics constitute another cause of antibiotic resistance. A study among the general public in the USA documented that people who have appropriate knowledge about antibiotics were more likely to complete a treatment course, however, they were also more likely to self-medicate and to keep leftover antibiotics for future use [7]. In 2000, the World Health Organization (WHO) report entitled “Overcoming Antimicrobial Resistance” identified three key issues with regards to public involvement: (1) improving access to medical services, (2) reducing unnecessary use of antimicrobial drugs and completing a full course of treatment, and (3) not sharing medication with other people or keeping part of the course for another occasion [8].

Nowadays, the high availability and increased use of antibiotics by the masses, which is not highly restricted in most developing countries, has resulted in the emergence of new multi-drug resistant pathogens [9]. Hence, there is a need to assess the knowledge of the general public, doctors, etc., through surveys about the prudent use of antibiotics to avoid further resistance [10]. Therefore, public awareness is an essential part of the solution to this issue.

A decent number of studies around the world about antibiotic knowledge and attitudes toward antibiotics targeting the general population have been published; however, only a few have been published targeting the general public of Saudi Arabia [11,12]. Hence, there is a need to examine the knowledge about antibiotic misuse among the general population of Saudi Arabia. Jeddah is the second largest city of Saudi Arabia and the major cosmopolitan city in the western part of the Kingdom. This study seeks to investigate the knowledge and attitudes among the general population in Jeddah toward antibiotics usage and could serve as baseline data and provide further insight to develop strategies for local health awareness.

## 2. Materials and Methods

### 2.1. Study Design, Area and Settings

The present study was conducted among the general population of Jeddah, Saudi Arabia, during the period from March till May 2018 at different shopping malls and public parks (five malls and two parks were visited on weekends). Self-administered questionnaires were distributed, and the participants filled out the survey directly on site. The researchers were present during the survey to answer any queries. The study was approved by the Institutional Review Board of King Abdullah International Medical Research Center, Jeddah, Saudi Arabia (Study No. SP17/441/J and Memo. Ref. No. IRBC/1626/17, approved 29 December 2017). Informed consent was obtained from each participant before data collection.

### 2.2. Sample Size Calculation

The sample size was computed using the Raosoft website, at the 95% confidence interval, with an estimated 50% response distribution and a margin of error of 5%. The required minimum sample size was determined to be 384. However, the final sample size was increased to 460 to account for a 20% non-response rate. A non-probability convenience sampling technique was used for selecting the study samples. There were no eligibility criteria for subjects to be included in the research, apart from their age being at least 18 years old.

### 2.3. Development of Questionnaire

The study questionnaire was adapted from a previous study and modified by the researchers to fit the study population [13]. The questionnaire consisted of four parts. Part 1 aimed to obtain the demographic characteristics of the general public. Part 2 aimed to assess recent usage of antibiotics among population for the past month. People were asked to provide additional information concerning the source and reason for taking, if they had taken, antibiotics in the past month. Part 3 included 14 statements subjects were asked to respond to in order to assess their knowledge of antibiotics. Statements included information about the purpose of antibiotics (five statements), identification of antibiotics (four statements), hazards of antibiotics (one statement for each: side effects, resistance, and allergic reactions), and completion of treatment courses (two statements). People were asked to select one of three options provided: “Yes”, “No”, or “Not Sure”. Part 4 consisted of nine statements designed to assess population attitudes toward antibiotics, including: antibiotics usage while suffering from colds, subjects’ expectations of doctors, consummation of treatment courses, sharing of antibiotics, retaining stocks of antibiotic for emergency use, leftover use of antibiotics, adhesion to antibiotics label instructions, reading the expiry date before taking antibiotics, and providing consultation to others suffering from colds.

In part 3 of the questionnaire, where population level of knowledge of antibiotics was assessed, questions were marked in a dichotomous manner. One point was given for each correct response and zero for each incorrect or unsure answer with the maximum attainable score being 14. A scoring system (0–14) to evaluate the level of knowledge was used based on given responses. The aggregate score of knowledge was classified into three levels betokened as good (10–14), moderate (5–9), and poor (0–4). A Likert-type scale was used to measure subjects’ attitudes in part 4, ranging from “Strongly Agree” to “Strongly Disagree.” Responses with “Agree” and “Strongly Agree” were considered as having agreed while “Disagree” and “Strongly Disagree” as having disagreed for the purpose of analysis simplicity. Positive responses/attitudes would denote the suitable use of antibiotics, whereas negative responses/attitudes would imply the inappropriate use of antibiotics. The options “Disagree” or “Strongly Disagree” for statements 1 to 6, and 9, and “Agree” or “Strongly Agree” for statements 7 and 8 indicated a positive response/attitude.

Reliability testing was performed in a previous study, which reflected a high internal consistency, with Cronbach’s α coefficient of 0.76 [13].

### 2.4. Data Analysis

Data were analyzed using the Statistical Package for Social Sciences (IBM Statistic, SPSS Inc., Chicago, IL, USA, version 20). Descriptive analysis was applied to sum up the data set (demographic characteristics, recent use of antibiotics, and knowledge and attitudes toward antibiotic usage). To test the influence of demographic characteristics on participants’ knowledge and attitude, chi-square or Fisher exact tests were applied wherever suitable. In addition, *p*-values were obtained for each test. The level of statistical significance was set at *p* < 0.05.

## 3. Results

The total number of distributed questionnaires to the general population of Jeddah was 460, 31 of which were returned incomplete (more than 50% of the questions were not answered) and, therefore, were excluded from the analysis (response rate of 93.26%). As described in the summary of demographic characteristics in Table 1, the age of more than half of participants (55.6%) was 18–30 years old. More than two third of participants were male (69.5%), while 131 were female (30.5%). More than half of participants (52.2%) were university educated, while 44.8% were secondary-school qualified. The monthly income of 29.9% was less than 3000 SAR, that of 24.8% was 3000–5000 SAR, while that of 30.2% was 5001–10,000 SAR, (SAR: Saudi Arabian Riyal (1 SAR = 0.27 USD)).

Regarding usage of antibiotics (Table 2), most participants (63.8%) reported not using antibiotics in the last month, while 36.2% recently used antibiotics. Regarding the source, almost half of those who used antibiotics (49%) did so without a prescription, having purchased them from a retail pharmacy (63.9%), private clinic (15.3%), or taken them from someone else’s supply (20.8%). The reasons for taking antibiotics were mostly because of fever, pain, and/or inflammation (58.2%), followed by respiratory illnesses (21.2%).

Almost half of participants (46.7%) showed moderate levels of knowledge regarding antibiotics as shown in Table 3, while 30.5% showed poor knowledge. Poor levels of knowledge about antibiotics were most frequent among participants aged 41–50 years (50%), while good knowledge about antibiotics was most frequent among those aged 18–30 years (29.8%) (Table 4). Participants’ levels of knowledge about antibiotics differed significantly according to their age groups (*p* = 0.003), but not their gender. Poor levels of knowledge were most frequent among those with intermediate educational status or lower (45.7%), while it was least frequent among those with university education (24.2%). Levels of knowledge were significantly associated with educational status (*p* = 0.029). Poor levels of knowledge were most frequent among those with monthly incomes 5001–1000 SAR (35.8%), while good levels of knowledge were most frequent among those with monthly incomes >10,000 SAR (35.8%), indicating a significant association between income and knowledge (*p* = 0.017).

Regarding the role of antibiotics (Table 5), the highest correct response was in identifying antibiotics as medicines that can kill bacteria (73.2%), with significant association to educational status (*p* < 0.001). However, 73.4% of participants incorrectly thought that antibiotics invariably relieve pain/inflammation, which was the highest incorrect response in the knowledge domain, and it was associated with age (*p* = 0.006) and educational status (*p* = 0.037). Age, educational status, and income were significant predictors for the statement “Antibiotics can be used to treat viral infections” (*p* = 0.017, *p* = 0.005, and *p* = 0.037 respectively). Education status also was significantly associated with the statement “Antibiotics are used to stop fever” (*p* = 0.006). As for the identification of antibiotics, more than half of respondents correctly responded that aspirin was not part of a new generation of antibiotics (56.3%) as compared to other medicines tested in this section. However, it is noteworthy that more than half of participants were unsure to whether diphenhydramine is an antibiotic (59.9%), with significant association to gender only (*p* = 0.038). Age was the only significant predictor for the statements “Aspirin is a new generation of antibiotic” and “Paracetamol is considered as an antibiotic” (*p* = 0.032 and *p* = 0.049, respectively). Regarding the dangers of antibiotics, more than half of participants had correct knowledge regarding all three knowledge items, with their income being the only significant predictor for the statement “All antibiotics do not cause side effects” (*p* = 0.003). Regarding completion of treatment courses, more than half of participants had correct knowledge regarding “The effectiveness of treatment is reduced if a full course of antibiotic is not completed” (52.1%). Income was the only significant predictor for the statement “You can stop taking a full course of antibiotics if your symptoms are improving” (*p* = 0.001).

In general, participants responded to most statements with negative attitudes (1, 2, 3, and 5), while responding to two statements with equal distribution (4 and 6), and two statements with positive attitudes (7 and 8), as shown in Table 6 (please refer to methodology section for the definition of positive and negative attitudes). Almost half of the respondents agreed they would take antibiotics when getting a cold (48.9%), expected antibiotics to be prescribed by their physician when suffering from common cold symptoms (45.2%), and would stop taking antibiotics when they started to feel well (45.5%). However, in statements 7 and 8, most participants had positive attitudes toward taking antibiotics according to the instruction on the label (71.8%) and looking at the expiry date of antibiotics before taking them (73.2%). Monthly income was the only significant predictor for three attitude statements. Two of the statements (1 and 3) significantly associated with monthly income had more negative attitudes from respondents than positive, (*p* = 0.037 and *p* = 0.021, respectively), whereas one statement (6) had an almost equal distribution in positive and negative attitudes (*p* = 0.042).

## 4. Discussion

In many countries, the distribution of antibiotics is not controlled by strict regulations. The practice of over-the-counter sales and use of antibiotics without physicians’ prescriptions has contributed to the issue of antibiotic resistance. Even though governmental regulations in Saudi Arabia indicate that antibiotics should be dispensed only upon physicians’ prescriptions, this is frequently ignored by pharmacies and the general public [14,15]. A systematic review found that the overall prevalence of people in Gulf Corporation Council (GCC) countries who had antibiotics without prescription ranged from 14% to 73% with the highest average prevalence in Saudi Arabia (55%), followed by Kuwait (28%), Oman (18%), and Qatar (14%) [16]. Our findings showed that the population of our study had variations in levels of knowledge regarding the purpose of antibiotics, especially regarding the treatment of pain/inflammation, fever, and all infections, including viral infections, with the percentage of those who had correct answers being 26.6%, 34.7%, 39.1%, and 45.4%, respectively. Findings of this study revealed that participants’ knowledge about antibiotics varied significantly based on several personal characteristics, age, educational status, and monthly income. On the other hand, participants’ attitudes toward antibiotics varied significantly based on their monthly income only. These findings are in accordance with those reported in a Malaysian study that found the general public’s age, educational level, and monthly income were significantly associated with their knowledge and attitudes toward antibiotic use [13].

Insufficient control over the availability of antibiotics could partially contribute to improper antibiotics use in the community. In some countries, people can obtain antibiotics without a medical prescription [17,18]. In a survey in Trinidad and Tobago, one fifth of the respondents obtained their antibiotics as over-the-counter medications at private pharmacies without any doctor’s prescription. Similarly, in Hong Kong, 9% of participants obtained antibiotics without a prescription [17,19]. On the other hand, a study in Al-Kharj City, Saudi Arabia, showed that more than half of the general public had obtained antibiotics without a prescription; 53.8% acquired their antibiotics from a retail pharmacy, and 3.6% from family members or friends [12,20].

The high percentage (49%) of antibiotic acquirement without prescription found in this study could be inaccurate, as this response is subjective. We expect that an even higher proportion of the general public have obtained antibiotics without prescriptions and consultations. This point necessitates further investigation to know the extent and relative importance of over-the-counter sales of antibiotics without prescriptions in Saudi Arabia. A European survey reported that the dispensing of antibiotics as only prescription medicines was somehow related to the lower rate of home-stored antibiotics and subsequently the misuse of antibiotics [21]. In a similar study, it was stressed that healthcare professionals should take a share in the responsibility for the misuse of antibiotics by the public [13].

The proportion of respondents in this study who thought that antibiotics were effective against viral infections (36.7%) was less than those reported in Europe (54–55%) and was much less than that reported by a survey conducted in the United States (70%) [17,22,23,24]. Responses to this statement were significantly associated with age, educational status, and income. This result could be due to the fact that around 52% of the participants in our study had a university education. The results in our study are also comparable to the systematic review, which revealed that 36–46% of participants from Gulf Corporation Council (GCC) countries did not know that antibiotics are not effective for viral infections or for colds (19–55%), but 25% to 52% believed that antibiotics can be used for coughs [16]. On the contrary, the statement with the highest incorrect response in this study was that the purpose of antibiotics was to relieve pain/inflammation (62.3%). Responses to this statement were significantly associated with age and educational status. This result is a higher percentage when compared to a study done in Malaysia assessing the knowledge and attitudes among the general public in regard to antibiotics use (51%) [13]. A possible reason for such a variation in knowledge could be due to improper counseling and/or provision of medical advice given by healthcare providers, leaving patients without a full understanding of their problem. Moreover, it was noted in this study that participants generally lacked the knowledge to differentiate between antibiotics and other commonly used medications. Further investigations are needed to gain more insight regarding this issue.

The findings of this study revealed that more than half of the participants (52.1%) had correct responses regarding the statement that effectiveness of treatment is reduced if a full course of antibiotics is not completed. Comparable proportions of respondents with correct knowledge were reported by other studies in Hong Kong (58%) and Taiwan (50.1%), while in Ethiopia, 92.1% agreed that the unnecessarily use of antibiotics can increase the resistance of bacteria [19,25,26]. In contrast, only 44.9% in this study correctly stated that they would continue with antibiotic treatment when even after they started feeling better. Similarly, while it was noted that only 38.2% of respondents realized the need to complete the course of antibiotics treatment, it was found that 30–72% of participants in GCC countries did not complete their antibiotic course as prescribed [16,27]. The main reasons given for not completing antibiotic courses were respondents feeling better and thinking that the used antibiotics did not work.

In this study, almost half of participants (48.9%) would take antibiotics for a cold to get better quickly. This finding is similar to the finding reported from a British community who thought antibiotics could relieve most cough and cold symptoms [28]. The proportion found in the present study is higher than those reported in the USA (27%), Hong Kong (17%), and Sydney, Australia (3%) [7,19,24,26,29]. In Baghdad, it was reported that the percentages of people who correctly answered that antibiotics have no role in managing viral infections, coughs or colds, and pain or inflammations were 42.4%, 20.0%, and 44.6%, respectively [27]. However, a recent study conducted in Saudi Arabia (Qassim region) documented findings similar to this study, with 75.2% of the participants thinking that the flu and common cold could be treated with antibiotics [30]. This reflects the immense gap in the awareness among the Saudi population regarding use of antibiotics in treating common colds and coughs.

Several studies indicated that frequent prescribing of antibiotics by physicians for viral respiratory infections has influenced public beliefs regarding the effectiveness of antibiotics in treating these illnesses [4,13,18]. In Ethiopia, it was reported that 83% of Ethiopians believed that antibiotics speed up the recovery of coughs and colds, while 78.4% believed that unnecessary use of antibiotics could increase bacterial resistance [26]. Subsequently, the misuse of antibiotics could be a repeated cycle that is detrimental in an era of increasing antibiotic resistance. Such misconceptions have contributed to the high expectations of antibiotic treatment for the common cold [13]. For our participants, the high expectations of antibiotic treatment for common cold symptoms (45.2%) are comparable with that of a study done in the United States, which reported that 48% of respondents would ask for antibiotics to treat cold symptoms [7]. However, lower percentages were reported in other studies around the world [19,22,25,29]. Moreover, it is to be noted that patients’ expectations constitute an important determinant of antibiotic prescription. Antibiotics are more likely to be prescribed under pressured in a clinical context [31]. However, inaccurate and over-estimation of patients’ expectations do occur, which results in unnecessary prescribing [32]. Interestingly, the decision to prescribe antibiotics could also be greatly influenced by the doctor-patient relationship in cases where the doctor would want to meet the patient’s expectations even when antibiotics were unnecessary for treatment. Nevertheless, some patients became satisfied with a better understanding of their illnesses even when antibiotics were not prescribed [32]. A recent study in southern Saudi Arabia (Aseer region) documented that the public in this region overall had poor knowledge regarding antibiotic use and recommended the importance of community awareness under the supervision of physicians [33].

## 5. Conclusions

About one third of the general public have poor knowledge about antibiotics and almost half of them have obtained antibiotics without a prescription. This could be a result of several common misconceptions among the general public, mainly related to the role of antibiotics and the importance of completing of antibiotic treatment courses. The results of our study indicate that there is room to improve the general public’s knowledge and to enhance their attitudes toward appropriate utilization of antibiotics. Moreover, the results of this study may lay the basis for planning well-organized educational programs from the healthcare system for the appropriate use of antibiotics by the healthcare system. This is the first study of the general public in Jeddah, Saudi Arabia, using an elaborated and validated questionnaire administered in a community setting. However, a higher sample size and probability sampling technique could increase the strength of further such studies. Furthermore, findings of this study cannot be generalized to the whole of Saudi Arabia.

## Figures and Tables

**Table 1:** Summary of demographic characteristics.

Characteristics	Number	Percentage (%)
Age (*n* = 427)		
18–30 years	237	55.5
31–40 years	114	26.7
41–50 years	46	10.8
51–60 years	22	5.1
>60 years	8	1.9
Gender (*n* = 429)		
Male	298	69.5
Female	131	30.5
Nationality (*n* = 428)		
Saudi	367	85.7
Non-Saudi	61	14.3
Highest educational status (*n* = 429)		
Primary or lower	13	3.1
Secondary	192	44.8
University	224	52.2
Monthly income (*n* = 352)		
<3000 SAR	105	29.9
3000–5000 SAR	87	24.8
5001–10,000 SAR	106	30.2
>10,000 SAR	54	15.1

SAR: Saudi Arabian Riyal (1 SAR = 0.27 USD); *n* differs because of incomplete answers in each subsection.

**Table 2:** Usage of antibiotics.

Recent Use (within 1 Month)	Number (*n* = 428)	Percentage (%)
Yes	155	36.2
No	273	63.8
Sources of antibiotics (*n* = 147)		
1. Prescription	75	51.0
2. Without prescription	72	49.0
a. Purchased from a private clinic	11	15.3
b. Purchased from a retail pharmacy	46	63.9
c. Used someone else’s antibiotics	15	20.8
Reasons for taking antibiotics		
Fever/Pain/Inflammation	96	61.9
Respiratory illness	35	22.6
Urinary tract infection	22	14.2
Skin problem/wound	12	7.7

**Table 3:** Levels of knowledge.

Level of Knowledge	Total Score	*n* (%)
Poor	0–4	131 (30.5)
Moderate	5–9	200 (46.7)
Good	10–14	98 (22.8)

**Table 4:** Participants’ levels of knowledge according to their demographic characteristics.

Characteristics	Level of Knowledge			*p* Value (χ^2^ Test)
	Poor (0–4)	Moderate (5–9)	Good (10–14)	
Age				
18–30 years	60 (25.3%)	109 (45.0%)	68 (29.7%)	**0.003**
31–40 years	33 (29.0%)	61 (53.5%)	20 (17.5%)	
41–50 years	23 (50.0%)	16 (34.8%)	7 (15.2%)	
51–60 years	10 (45.5%)	11 (50.0%)	1 (4.5%)	
>60 years	3 (37.5%)	3 (37.5%)	2 (25.0%)	
Gender				
Male	94 (31.5%)	132 (44.3%)	72 (24.2%)	0.383
Female	37 (28.2%)	68 (51.9%)	26 (19.9%)	
Educational status				
Intermediate or lower	5 (38.5%)	6 (46.1%)	2 (15.4%)	**0.029**
Secondary	64 (33.3%)	97 (50.5%)	31 (16.2%)	
University	62 (27.7%)	97 (43.3%)	65 (29.0%)	
Monthly income				
<3000 SAR	35 (33.3%)	55 (52.4%)	15 (14.3%)	**0.017**
3000–5000 SAR	20 (23.0%)	42 (48.3%)	25 (28.7%)	
5001–10,000 SAR	38 (35.9%)	44 (41.5%)	24 (22.6%)	
>10,000 SAR	18 (33.3%)	16 (29.7%)	20 (37.0%)	

The level of statistical significance was set at *p* < 0.05.

**Table 5:** Association of demographic characteristics with knowledge statements.

Statement	Correct Answer	Incorrect Answer	Unsure	*p* Value (X^2^ Test/Fisher Exact Test)			
				Age	Gender	Educational Status	Income
Role of Antibiotics							
Antibiotics are medicines that can kill bacteria.	312 (73.2%)	46 (10.8%)	68 (16.0%)	**0.000**	1.000	**0.000**	0.975
Antibiotics can be used to treat viral infections.	193 (45.4%)	156 (36.7%)	76 (17.9%)	0.017 *	0.285	**0.005**	**0.037**
Antibiotics can cure all infections.	167 (39.1%)	149 (34.9%)	111 (26.0%)	0.069	0.291	0.200	**0.044**
Antibiotics are indicated to relieve pain/inflammation.	113 (26.6%)	265 (62.3%)	47 (11.1%)	0.006 *	0.813	**0.037**	0.182
Antibiotics are used to stop fever.	148 (34.7%)	205 (48.2%)	73 (17.1%)	0.218 *	0.485	**0.006**	0.654
Identification of Antibiotics							
Penicillin is an antibiotic.	205 (48.4%)	71 (16.7%)	148 (34.9%)	**0.000**	0.755	0.657	0.264
Aspirin is a new generation of antibiotic.	240 (56.3%)	57 (13.4%)	129 (30.3%)	0.032 *	0.616	**0.057**	0.116
Paracetamol is considered as an antibiotic.	202 (47.5%)	86 (20.2%)	137 (32.3%)	0.049 *	0.729	0.600	**0.037**
Diphenhydramine is not an antibiotic.	73 (17.2%)	97 (22.9%)	254 (59.9%)	0.056	**0.038**	0.526	0.289
Dangers of Antibiotics							
Overuse of antibiotics can cause antibiotic resistance.	242 (56.9%)	99 (23.3%)	84 (19.8%)	0.438	0.692	0.056	0.651
Antibiotics may cause allergic reaction.	236 (55.7%)	66 (15.5%)	122 (28.8%)	0.109 *	0.070	0.175	0.581
All antibiotics do not cause side effects.	243 (56.9%)	107 (25.1%)	77 (18.0%)	**0.000**	0.289	0.473	**0.003**
Completion of Treatment Courses							
You can stop taking a full course of antibiotic if your symptoms are improving.	191 (44.9%)	170 (40.0%)	64 (15.1%)	0.183	0.728	0.119	**0.001**
The effectiveness of treatment is reduced if a full course of antibiotic is not completed.	222 (52.1%)	116 (27.2%)	88 (20.7%)	0.563 *	0.387	0.190	0.307

The level of statistical significance was set at *p* < 0.05. *: Fisher exact test.

**Table 6:** Association of demographic characteristics with attitude statements.

Statement	Agree	Disagree	Unsure	*p* Value (X^2^ Test/Fisher Exact Test)			
				Age	Gender	Educational Status	Income
1. When I get a cold, I will take antibiotics to help me get better more quickly.	208 (48.9%)	152 (35.8%)	65 (15.3%)	**0.014**	0.366	0.679	**0.037**
2. I expect antibiotics to be prescribed by my doctor if I suffer from common cold symptoms	191 (45.2%)	89 (21.0%)	143 (33.8%)	0.065 *	0.083	0.994	0.110
3. I normally stop taking antibiotics when I start feeling better.	191 (45.5%)	147 (35.0%)	82 (19.5%)	0.261 *	0.970	0.648	**0.021**
4. If my family member is sick, I usually will give my antibiotics to them.	107 (25.2%)	208 (48.9%)	110 (25.9%)	**0.038**	0.294	0.357	0.133
5. I normally keep antibiotics stocks at home in case of emergency.	158 (37.5%)	162 (38.5%)	101 (24.0%)	**0.013**	0.673	0.064 *	0.051
6. I will use leftover antibiotics for a respiratory illness.	98 (23.2%)	374 (46.1%)	130 (30.7%)	0.123 *	0.298	0.370	0.042
7. I will take antibiotics according to the instructions on the label *.	305 (71.8%)	34 (8.0%)	86 (20.2%)	0.233 *	0.658	0.406	0.759
8. I normally will look at the expiry date of antibiotics before taking them *.	311 (73.2%)	26 (6.1%)	88 (20.7%)	0.072 *	0.092	0.757	0.690

The level of statistical significance was set at *p* < 0.05. *: Fisher exact test.

## Data Availability

The datasets generated or analyzed during the study are available with the corresponding author on acceptable request.
